# ТТГ-продуцирующая микроаденома гипофиза: проблемы диагностики в дебюте заболевания

**DOI:** 10.14341/probl12860

**Published:** 2022-03-09

**Authors:** А. В. Климчук, И. А. Яцков, К. В. Бублей, Д. А. Энзель, А. С. Щербаков

**Affiliations:** Крымский федеральный университет им. В.И. Вернадского; Республиканская клиническая больница им. Н.А. Семашко; Крымский федеральный университет им. В.И. Вернадского; Крымский федеральный университет им. В.И. Вернадского; Крымский федеральный университет им. В.И. Вернадского; Крымский федеральный университет им. В.И. Вернадского

**Keywords:** микроаденома гипофиза, тиреотропинома, ТТГ-продуцирующая аденома, многоузловой зоб, тиреотоксикоз

## Abstract

Опухоли гипофиза, продуцирующие тиреотропный гормон (ТТГ), встречаются редко и составляют около 1–3% всех аденом гипофиза, наиболее часто наблюдаются у лиц молодого и трудоспособного возраста. Статья представляет клинический случай тиреотропиномы, которая была диагностирована через 6 лет после первичного обращения к эндокринологу у 44-летней женщины. В дебюте заболевания тиреотропинома проявила себя изолированным подъемом уровня ТТГ при нормальных уровнях свободного тироксина (Т4) и свободного трийодтиронина (Т3). Больная постоянно принимала β-блокаторы из-за беспокоящей ее тахикардии. Поставлен диагноз: субклинический гипотиреоз, по поводу чего она периодически наблюдалась, контролируя уровень ТТГ и принимая препараты левотироксина в дозе до 175 мкг, что сопровождалось повышенным уровнем ТТГ. Через 6 лет выявлено повышение уровней, кроме ТТГ, свободного Т3 и свободного Т4. На магнитно-резонансной томографии с внутривенным контрастным усилением обнаружена микроаденома гипофиза размером 4 мм, при лабораторном исследовании всех тропных гормонов выявлено изолированное повышение ТТГ. При диагностировании ТТГ-продуцирующей аденомы гипофиза выполнена транссфеноидальная аденомэктомия. За время 3-летнего послеоперационного наблюдения рецидив аденомы отсутствует, развился вторичный гипотиреоз, больная в настоящий момент постоянно принимает левотироксин 75 мкг в сутки.

## Abstract (en)

Tumors of the pituitary gland producing thyrotropin hormone (TSH) are rare and account for about 1–3% of all pituitary adenomas, most frequently occurring in persons of young and working age. This article presents a clinical case of thyrotropinoma in a 44-year-old woman, which was diagnosed 6 years after her initial visit to an endocrinologist. At the debut of the disease, thyrotropinoma manifested as an isolated elevation of TSH, with normal levels of free T4 and free T3. The patient was constantly taking β-blockers due to her disturbing tachycardia. She was diagnosed with subclinical hypothyroidism, for which she was periodically observed, controlling her TSH level and taking levothyroxine drugs in a dose of up to 175 mcg, which was accompanied by elevated TSH levels. After 6 years, there was an increase in free T3 and free T4 in addition to TSH. Magnetic resonance imaging with intravenous contrast enhancement revealed a pituitary microadenoma 4 mm in size, and laboratory examination of all tropic hormones revealed an isolated increase in TSH. Transsphenoidal adenomectomy was performed at the diagnosis of TSH-producing pituitary adenoma. During the three-year postoperative follow-up there was no adenoma recurrence, secondary hypothyroidism developed, the patient currently takes levothyroxine 75 mcg per day continuously

## АКТУАЛЬНОСТЬ

Опухоли гипофиза составляют 14% первичных опухолей внутричерепной области и центральной нервной системы. Согласно статистическим данным, ежегодно в России и странах СНГ выявляется около 3 тыс. человек с данной патологией [1][2]. Известно, что часть опухолей гипофиза (около 36–54%) не секретируют гормоны, а примерно половина из них (46–64%) являются гормонопродуцирующими. Аденомы гипофиза чаще выявляются у лиц молодого и трудоспособного возраста и нередко приводят к инвалидизации этих больных. Наиболее часто встречаются аденомы гипофиза, секретирующие пролактин (32–51%), гормон роста (9–11%) и адренокортикотропный гормон (3–6%) [3]. Опухоли гипофиза, продуцирующие изолированно тиреотропный гормон (ТТГ), встречаются редко и составляют около 1–3% всех его аденом [4–6]. Тиреотропиномы гипофиза в большинстве выявленных случаев (82–87%) представляют собой макроаденомы. Однако за последние 5 лет отмечается учащение выявления ТТГ-продуцирующих микроаденом гипофиза [7][8]. Этому способствует использование в настоящее время сверхчувствительного иммунометрического анализа ТТГ и магнитно-резонансной томографии (МРТ) для визуализации [9].

С целью акцентирования внимания эндокринологов на этой патологии мы представляем клинический случай тиреотропиномы у 44-летней женщины, диагностирование аденомы гипофиза у которой произошло через 6 лет после ее первичного обращения к специалисту.

## ОПИСАНИЕ СЛУЧАЯ

Больная П., 44 лет обратилась к эндокринологу поликлиники ГБУЗ РК «РКБ им. Н.А. Семашко» г. Симферополя с жалобами на постоянное сердцебиение, частые тупые умеренно выраженные головные боли, потливость, ощущение жара, раздражительность, утомляемость, общую слабость, бессонницу.

Из анамнеза известно, что вышеперечисленные жалобы, а также снижение массы тела на 5 кг за 1 мес появились у пациентки примерно 6 лет назад, с чем она впервые обратилась к эндокринологу. На тот момент при обследовании у пациентки было выявлено повышение показателей: ТТГ — 15 мкМЕ/мл (норма 0,2–4,2), антитела к тиреоглобулину — 808,2 МЕ/мл (норма 0–115 МЕ/мл), антитела к тиреопероксидазе — 448,7 МЕ/мл (норма 0–34 МЕ/мл) и нормальный уровень свободного тироксина (сТ4) — 15,4 пмоль/л (норма 10,8–22,0), глюкоза крови — 4,4 ммоль/л. УЗИ щитовидной железы: общий объем железы 24,3 см3. Обе ее доли повышенной эхогенности, диффузно-неоднородной структуры за счет обширных участков повышенной и сниженной эхогенности, васкуляризация железы повышена, внутриорганные сосуды не расширены, лимфоузлы не изменены. Заключение УЗИ щитовидной железы: отмечаются эхо-признаки гипертрофии щитовидной железы и аутоиммунного тиреоидита. Остальные общеклинические и биохимические исследования были без патологических изменений. Больной был поставлен диагноз субклинического гипотиреоза, назначен левотироксин в дозе 25 мкг/сут. Рекомендован контроль ТТГ каждые 3–6 мес. Пациентка выполняла назначения врача. После постановки диагноза и начала лечения гормоны щитовидной железы (свободный трийодтиронин (сТ3), сТ4) не контролировались. На фоне лечения — вес стабильный, беспокоили тахикардия, нарушение сна, общая слабость. Несмотря на постоянное постепенное увеличение дозы левотироксина от 25 до 150 мкг/сут, подавления ТТГ не наблюдалось. Уровень ТТГ находился в диапазоне 5,6–8,5 мкМЕ/мл (норма 0,2–4,2).

Через 5 лет больная обратилась к другому специалисту, так как ее продолжали беспокоить жалобы на учащенное сердцебиение, слабость, нарушение сна, периодическое повышение артериального давления до 160/80 мм рт. ст., частые умеренно выраженные головные боли, а также отсутствие нормализации уровня ТТГ на фоне лечения левотироксином. На этот момент пациентка принимала левотироксин 150 мкг/сут. При обследовании выявлен повышенный уровень ТТГ — 4,9 мкМЕ/мл (норма 0,4–4,0). На УЗИ щитовидная железа диффузно-неоднородная, эхогенность повышена. В правой доле в среднем отделе визуализируется гиперэхогенный узел диаметром 10 мм с периферическим кровотоком. В нижнем отделе определяется аналогичный узел диаметром 10,5 мм. Общий объем железы: 19 см3. Васкуляризация железы повышена, внутриорганные сосуды не расширены. Лимфоузлы не изменены. В заключении: УЗ-признаки аутоиммунного тиреоидита, узлового зоба. Общеклинические и биохимические анализы без патологических изменений. Поставлен диагноз: Аутоиммунный тиреоидит, гипотиреоз. Увеличена доза левотироксина до 175 мкг/сут, назначен бисопролол 2,5 мг/сут.

Через 2 мес, на фоне получаемого лечения, больную по-прежнему беспокоят сердцебиение и вышеперечисленные жалобы. При обследовании гормонального статуса выявлено повышение уровней ТТГ — 5,1 мкМЕ/мл (норма 0,2–4,2), сТ4 — 3,7 нг/дл (норма 0,9–1,7), сТ3 — 6,7 нг/дл (норма 2,0–4,4). Выполнена МРТ головного мозга. При МРТ-сканировании межполушарная щель находится строго посредине. Признаков объемного воздействия на структуры мозга не выявлено. Серое и белое вещество полушарий головного мозга нормально развито. В переднем полюсе правой височной доли определяется единичный очаг округлой формы, гиперинтенсивный, размером 4 мм, без перифокального отека и масс-эффекта. Базальные ганглии, внутренняя капсула, мозолистое тело и таламус выглядят неизмененными. Турецкое седло и параселлярные структуры без видимых изменений. Гипофиз без видимой патологии, воронка не смещена. Заключение: МРТ-признаки единичного очагового изменения правой височной доли. В гипофизе патологических образований не выявлено. Поставлен диагноз: Аутоиммунный тиреоидит, гипотиреоз. Рекомендовано снизить дозу левотироксина до 150 мкг/сут с контролем ТТГ через 3 мес, продолжать прием бисопролола 2,5 мг/сут.

Пациентка вредные привычки, в том числе курение, отрицает. Семейный анамнез не отягощен заболеваниями гипофиза, щитовидной железы и другими эндокринными заболеваниями. У матери — острое нарушение мозгового кровообращения в 40 лет. Из сопутствующих заболеваний: хронический холецистит, киста правой гайморовой пазухи. Гинекологический анамнез: в юности отмечались нарушения менструального цикла, в течение 1 года была аменорея, лечилась по поводу бесплодия 2-й степени; на фоне лечения кломифеном менструальный цикл восстановился, 2 беременности: в 2000 г. — роды, в 2006 г. — кесарево сечение. В настоящий момент — миома матки малых размеров. Операция по поводу аденофлегмоны шеи (после травмы головы) в детстве. Аллергия на новокаин.

На момент обращения больная принимала левотироксин 150 мкг/сут. Объективно. Общее состояние удовлетворительное. Вес 57 кг. Рост 168 см. Индекс массы тела 20,2 кг/м2 (норма). Температура 36,8 °С. Кожные покровы чистые, естественной окраски, теплые, умеренно влажные. Видимые слизистые оболочки чистые, обычной окраски. Глазные симптомы — отрицательные. Слабовыраженный тремор рук в позе Ромберга. Щитовидная железа при пальпации эластична, подвижна, безболезненная, несколько увеличена за счет обеих долей. Регионарные и периферические лимфоузлы не увеличены. Тоны сердца приглушены, ритмичны, шумы отсутствуют. Артериальное давление 140/85 мм рт. ст. Частота сердечных сокращений 88 в минуту. Дыхание везикулярное, хрипов нет. Число дыхательных движений — 16 в 1 мин. Язык влажный, живот при поверхностной пальпации мягкий, безболезненный во всех отделах, печень у края реберной дуги, селезенка не увеличена. Симптом поколачивания по поясничной области отрицательный с обеих сторон, Мочеиспускание свободное, безболезненное, стул, со слов, регулярный. Отеков нет.

Результаты физикального, лабораторного и инструментального исследования

Уровни гормонов при обращении на фоне приема левотироксина 150 мкг/сут: отмечаются повышение ТТГ — 6,7 мкМЕ/мл (норма 0,2–4,2), сТ4 — 33,3 пмоль/л (норма 10,8–22,0), сТ3 — 10,3 пмоль/л (норма 3,1–6,3) и нормальный уровень пролактина 252,8 мЕд/л (норма 64–395).

УЗИ щитовидной железы.

Контуры: четкие, ровные. Правая доля, размеры: длина — 6,0 см, ширина — 2,0 см, толщина — 1,8 см; объем — 10,3 см3. Левая доля, размеры: длина — 5,4 см, ширина — 1,9 см, толщина — 1,8 см; объем — 8,8 см3. Общий объем щитовидной железы: 19,1 см3. Перешеек: 0,3 см. Васкуляризация при цветовом допплеровском картировании (ЦДК): умеренно усилена. Расположение: обычное. Структура: неоднородная. Эхогенность: понижена. Объемное образование в средней трети по задней поверхности — образование средней эхогенности с четкими контурами диаметром 0,9 см, в нижней трети — аналогичное образование размеры 1,4×1,3×1,0 см. Регионарные лимфатические узлы: с обеих сторон — паратрахеальные лимфоузлы до 0,9×0,5 см, однородной структуры. Заключение: эхографические признаки аутоиммунного поражения щитовидной железы в сочетании с правосторонним многоузловым зобом. TIRADS 2.

Гистологические и цитологические исследования пунктата щитовидной железы.

Результат цитологического исследования: пунктирован узел коллоидного в разной степени пролиферирующего зоба с очагами оксифильноклеточной трансформации тиреоцитов, регрессивными изменениями, лимфоидной инфильтрацией (по Bethesda Thyroid Classification категория II).

ЭКГ.

Ритм: синусовый с ЧСС 69 в минуту. Положение электрической оси сердца: вертикальное. Изменение предсердного компонента (перегрузка левого предсердия). Признаки перегрузки левого желудочка. Особенности в/желудочковой проводимости по правой ножке пучка Гиса.

Консультация офтальмолога.

Заключение: Ангиопатия сетчатки по гипертоническому типу обоих глаз.

Больной рекомендовано продолжать прием бисопролола 2,5 мг/сут, отменить левотироксин. Пациентка отказалась отменять левотироксин, объясняя это тем, что принимает препарат 6 лет и боится ухудшения самочувствия после его отмены. Больной рекомендовано снизить суточную дозу левотироксина до ¼ таблетки (37,5 мкг) и повторить гормональные исследования через 2 нед. При повторном обследовании выявлены повышение ТТГ — 7,0 мкМЕ/мл (норма 0,2–4,2), сТ4 — 25,9 пмоль/л (норма 10,8–22,0), сТ3 — 7,1 пмоль/л (норма 3,1–6,3) и нормальный уровень пролактина — 237,2 мкМЕ/мл (норма 64–395). После получения результатов повторно рекомендовано отменить левотироксин, продолжать прием бисопролола 2,5 мг/сут. Больная выполняет рекомендации. Через 2 мес после отмены левотироксина при исследовании отмечалось повышение ТТГ — 8,9 мкМЕ/мл (норма 0,2–4,2), сТ4 — 23,7 пмоль/л (норма 10,8–22,0), сТ3 — 9,1 пмоль/л (норма 3,1–6,3).

Больной рекомендована МРТ гипофиза с внутривенным контрастным усилением. Результат МPT головного мозга с внутривенным контрастным усилением (15,0 омнискан): на серии томограмм получены изображения суб- и супратенториальных структур головного мозга. В режимах T1W, T2 FLAIR и T2W патологических МР-сигналов от вещества мозга не получено. Признаков объемного образования не выявлено. Срединные структуры симметричны, не смещены. Базальные цистерны обычной конфигурации, не расширены. После внутривенного усиления гипофиз неоднородно выполнен контрастом, в левой половине гипофиза определяется микроаденома размером 4 мм в диаметре. Ножка гипофиза не отклонена. Заключение: Микроаденома гипофиза (рис. 1).

**Figure:**
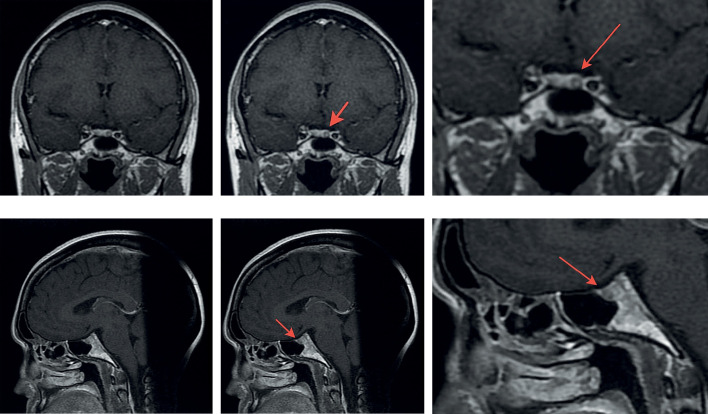
Рисунок 1. Магнитно-резонансная томография хиазмально-селлярной области. Выделена микроаденома гипофиза.

При исследовании тропных гормонов гипофиза выявлены повышение уровня ТТГ — 7,7 мкМЕ/мл (норма 0,2–4,2) и нормальные уровни соматотропного гормона — 12 мЕд/л (норма 0,2–13 мЕд/л), фолликулостимулирующего гормона — 13,8 мЕд/мл (норма 3,4–21,6 мЕд/мл), инсулиноподобного фактора роста 1 — 239,4 нг/мл (норма 82–293). Отмечалось повышение уровня пролактина 486,2 мЕд/л (норма 64–395) при нормальном уровне биоактивного пролактина 206 мЕд/л (норма 64–395). Выявлено повышение гормонов щитовидной железы: сТ4 — 24,9 пмоль/л (норма 10,8–22,0), сТ3 — 8,7 пмоль/л (норма 3,1–6,3) и нормальный уровень антител к рецепторам ТТГ — 0,5 МЕ/л (норма 0–1,8).

Больной поставлен диагноз: ТТГ-секретирующая микроаденома гипофиза. Узловой зоб. Ангиопатия сетчатки по гипертоническому типу обоих глаз. Пациентка направлена в ФГБУ «НМИЦ эндокринологии», г. Москва, где диагноз был подтвержден, проведено хирургическое лечение — транссфеноидальная аденомэктомия.

Морфологическое исследование послеоперационного материала гипофиза.

Микроскопическое описание: фрагменты аденомы гипофиза из хромофобных клеток, с 2 митозами, солидного строения. Заключение: аденома гипофиза. Код МКБ: D35.2 Доброкачественное новообразование гипофиза.

Исход и результаты последующего наблюдения

В настоящий момент прошло 3 года после оперативного лечения. Больная жалоб не предъявляет. Общее состояние удовлетворительное. Наблюдается эндокринологом по поводу вторичного гипотиреоза, многоузлового зоба. Пациентка получает постоянно левотироксин 75 мкг/сут. Уровни гормонов в норме: сТ3 — 3,7 пг/мл (норма 2,5–4,3), сТ4 — 1,3 нг/дл (норма 0,9–1,7), ТТГ — 1,8 мкМЕ/мл (норма 0,3–4,2).

MPT головного мозга с внутривенным усилением (15,0 омнискан): на серии томограмм получены изображения суб- и супратенториальных структур головного мозга. Состояние после оперативного лечения (удаления микроаденомы гипофиза). После внутривенного усиления гипофиз однородно гомогенно накапливает контрастное вещество. Ножка гипофиза не отклонена. В режимах TIW, T2 FLAIR и T2W патологических МP-сигналов от вещества мозга не получено. Признаков объемного образования не выявлено. Заключение: органических изменений головного мозга не выявлено.

УЗИ щитовидной железы.

Перешеек: 2,3 мм. Расположение типичное. Контуры четкие. Правая доля: 46×16×17 мм. Объем: 6,5 см3. Эхогенность смешанная, больше средняя. Эхоструктура неоднородная за счет наличия двух изоэхогенных узловых образований с четкими ровными контурами, с гипоэхогенным ободком в средней трети у задней стенки 7,7×6,5×7 мм и в нижнем полюсе 12×7,6×10 мм с умеренным перинодулярным кровотоком при ЦДК в обоих узлах; за счет наличия множества гипоэхогенных зон неправильной и в меньшем количестве овальной формы. Левая доля: 47×15×15 мм. Объем: 5,5 см3. Эхогенность смешанная, больше средняя. Эхоструктура неоднородная за счет наличия множества гипоэхогенных зон неправильной и в меньшем количестве овальной формы. Общий объем: 11,0 см3. Кровоток в обеих долях в режиме ЦДК — диффузно повышен. Лимфатические узлы по ходу общей сонной артерии с обеих сторон — без патологических изменений. Заключение: УЗ-признаки аутоиммунного тиреоидита, узлового зоба.

## ОБСУЖДЕНИЕ

Редкость данной патологии, слабо выраженная клиническая картина центрального тиреотоксикоза на фоне приема β-блокаторов, отсутствие прогрессирования заболевания в описанном нами клиническом случае привели к позднему выявлению микроаденомы гипофиза: от появления клинических симптомов и обращения больной к врачу до диагностирования тиреотропиномы прошло 6 лет.

В дебюте заболевания у данной пациентки тиреотропинома проявила себя изолированным повышением ТТГ на фоне нормального уровня гормонов сТ3, сТ4, что было расценено специалистом как «субклинический гипотиреоз». В дальнейшем лечение и наблюдение больной проходило как пациентки с гипотиреозом: контролировался уровень ТТГ на фоне непрерывного наращивания дозы левотироксина. Однако при заместительной гормонотерапии, несмотря на увеличение дозы левотироксина до 175 мг, подавления ТТГ не отмечалось. Обращает на себя внимание не только отсутствие достижения нормальных уровней ТТГ на фоне проводимого лечения в течение нескольких лет, но и наличие жалоб больной на чувство жара, нарушение сна, сердцебиение, эмоциональную лабильность, частые умеренные головные боли. Вышеперечисленное может служить поводом для специалистов для более широкого обследования таких пациентов и периодического контроля профилей гормонов щитовидной железы сТ3, сТ4 в комбинации с ТТГ для исключения или выявления ТТГ-продуцирующей аденомы.

В представленном нами случае у пациентки отмечался клинически умеренно выраженный тиреотоксикоз, даже несмотря на постоянный прием препаратов левотироксина. Это можно объяснить постоянным приемом β-блокаторов пациенткой, а также соответствует описанным ранее в литературных источниках случаям тиреотропином, где отмечалось, что клинические проявления тиреотоксикоза центрального генеза менее выражены, чем у пациентов с болезнью Грейвса [10].

Уровень ТТГ у больной за весь период заболевания колебался в диапазоне от 4,9 до 15,5 мкМЕ/мл. Это соответствует существующим литературным данным, что ТТГ у пациентов с тиреотропиномами часто может находиться в пределах от субнормальных до слегка повышенных значений — около 10 мкМЕ /мл [9].

В периодической литературе встречается описание клинического случая чрезвычайно высокого уровня ТТГ (288,2 мкМЕ/мл) у пациента с тиреотропиномой и тяжелым гипотиреозом. В этой ситуации заместительная гормонотерапия привела к снижению опухолевой массы гипофиза [11]. Можно предположить, что в этом случае причиной возникновения ТТГ-продуцирующей аденомы у больного явился длительно существующий тяжелый гипотиреоз, приведший к долго существующей продукции высокого уровня ТТГ. Вероятно, поэтому назначение левотироксина привело к улучшению состояния этого пациента и уменьшению аденомы гипофиза. В нашем клиническом случае такого эффекта от приема больной данного препарата не наблюдалось.

В представленном нами клиническом случае у пациентки на фоне долго существующего высокого уровня ТТГ отмечалось увеличение объема щитовидной железы, а также появление в ней узлов. Это соответствует литературным данным, описывающим наличие узлового или многоузлового зоба у большинства больных с тиреотропиномой. Иногда его выявление у этой категории больных при наличии выраженных клинических проявлений тиреотоксикоза и отсутствии правильной диагностики приводит к тиреоидэктомии, что, в свою очередь, ведет к значительному увеличению опухолевой массы гипофиза, а часто и к рецидивированию многоузлового зоба, если выполнена частичная резекция щитовидной железы [12][13].

Ранее существующие наблюдения позволили сделать вывод, что тиреотропиномы — медленнорастущие опухоли гипофиза [14][15]. В пользу этого утверждения говорит и наш клинический случай, в котором, несмотря на существование ТТГ-продуцируемой аденомы около 6 лет, нарушений полей зрения и больших размеров аденомы у пациентки не отмечалось.

Клиническая значимость раннего выявления данного редкого заболевания несомненна, но имеет определенные диагностические и терапевтические трудности. Неспособность вовремя распознать тиреотропиному может привести к нежелательным последствиям, таким как ошибочное удаление щитовидной железы у пациентов с центральным гипертиреозом, а гипердиагностика заболевания — к операциям на гипофизе у пациентов с резистентностью к гормонам щитовидной железы. Ранняя диагностика и лечение ТТГ-продуцирующей аденомы гипофиза могут предотвратить возникновение таких осложнений, как дефекты зрения из-за сдавления хиазмы зрительного нерва, развитие гипопитуитаризма. При выполнении нашей пациентке первичной МРТ головного мозга без внутривенного контрастного усиления микроаденома гипофиза не выявлена, что отсрочило время постановки правильного диагноза.

По данным исследования E. Malchiodi и соавт., наблюдавших 68 больных после проведенной транссфеноидальной аденомэктомии по поводу тиреотропиномы, рецидивы заболевания в этой категории пациентов редки и составляют 3% случаев, недостаточность других тропных гормонов после операции развивается у 9% больных [16]. Трехлетнее наблюдение пациентки после выполненной транссфеноидальной аденомэктомии показало отсутствие рецидива заболевания и нарушений со стороны секреции других тропных гормонов гипофиза. Больная в настоящий момент не имеет жалоб и отмечает значительное улучшение самочувствия после проведенного оперативного лечения. Своевременное выявление и лечение этой категории больных значительно улучшают их качество жизни и прогноз заболевания.

Приведенный клинический случай должен обратить внимание эндокринологов на проблемы ранней диагностики тиреотропином.

## ЗАКЛЮЧЕНИЕ

1. Тиреотропинома в дебюте заболевания проявляет себя изолированным повышением ТТГ на фоне нормального уровня гормонов щитовидной железы сТ3, сТ4.

2. Клинические проявления центрального гипертиреоза у пациентов с тиреотропиномой слабо выражены при приеме β-блокаторов.

3. При микроаденоме гипофиза МРТ без внутривенного контрастного усиления дает ложноотрицательный результат.

## ДОПОЛНИТЕЛЬНАЯ ИНФОРМАЦИЯ

Источники финансирования. Работа выполнена по инициативе авторов без привлечения финансирования.

Конфликт интересов. Авторы декларируют отсутствие явных и потенциальных конфликтов интересов, связанных с содержанием настоящей статьи.

Участие авторов. Климчук А.В. — концепция статьи, работа с пациентом, анализ публикаций, интерпретация результатов, внесение существенных правок с целью повышения научной ценности статьи; Яцков И.А. — анализ публикаций, интерпретация результатов, внесение существенных правок с целью повышения научной ценности статьи; Бублей К.В. — сбор и систематизация данных, написание статьи; Энзель Д.А. — сбор и систематизация данных, написание статьи; Щербаков А.С. — сбор и систематизация данных, написание статьи. Все авторы одобрили финальную версию статьи перед публикацией, выразили согласие нести ответственность за все аспекты работы, подразумевающую надлежащее изучение и решение вопросов, связанных с точностью или добросовестностью любой части работы.

Согласие пациента. Пациент добровольно подписал информированное согласие на публикацию персональной медицинской информации в обезличенной форме.
